# Vagueness and Ambiguity in Communication of Case Management: A Content Analysis in the Australian National Disability Insurance Scheme

**DOI:** 10.5334/ijic.5590

**Published:** 2021-03-19

**Authors:** Sue Lukersmith, Julia Taylor, Luis Salvador-Carulla

**Affiliations:** 1 Australian National University, Australia; 2 University of Sydney, Australia

**Keywords:** case management, care coordination, support coordination, National Disability Insurance Scheme, CMTaxonomy, disability, integrated care

## Abstract

Case management (CM) is an integrated care strategy, characterised by a set of actions to support person-centred planning, coordination of health and social services. Decades of CM, organisational psychology and occupational research highlight how vagueness and ambiguity in role communication can create role conflict and job stress, negatively impacts staff turnover, intra-organisational collaboration, job performance, and that poor communication of CM impedes policy, quality analysis service development and practice. We conducted a detailed top-down hierarchical, quality analysis of communication about CM roles and responsibilities in a Scheme for people with disability in Australia. The study used content analysis methods and the main actions as defined in a validated CM taxonomy (Appendix 1). We systematically searched and analysed 53 Scheme policy and practice documents of CM from 2013–2019. The results showed poor role communication with vagueness, ambiguity, gaps in the description of CM roles and responsibilities. Poor role communication has contributed to negative experiences and outcomes of CM actions of planning and coordination, as reported by CM users in many Scheme-related parliamentary inquiries, research, formal complaints, and decision appeals. The results reinforce the importance of an ontological approach in communication of CM roles and actions and provides learnings for integrated care roles across countries and contexts.

## Introduction

Case management (CM) makes a unique contribution to the integration of community-based care and multiple sectors supports, to support the person and achieve their goals [1,2,3]. CM differs from system-based integration (or coordination by professionals across their services), because of the relationship the case manager has with the user. In both literature and practice the term CM and care coordination are used synonymously for both relational CM and system-based care coordination. In this paper we refer only to CM where there is a ‘relational continuity’ and a continuous (professional) relationship between the case manager and user (p.6 [4]).

CM effectiveness is demonstrated for planning and coordination of health and social support services, and positively impacts on individual outcomes, social and economic participation [5,6,7,8]. Despite this, over many years of CM quality, evaluation studies and systematic reviews in different countries, contexts, and health conditions, were beset with difficulties. The variability and complexity of CM is often underscored as a key issue, yet the value and adaptability of CM interventions is highlighted [9,10]. Overwhelmingly, authors conclude the key difficulties are due to inconsistencies and ambiguity in definitions, vague descriptions in communication and lack of a common language for CM [11,12,13,14]. The high variability occurs with the factors of context, service setting, health condition, model, position title, discipline and skills of the case manager and actions performed [15]. Vague and ambiguous communication on CM is highly problematic for policy, service planning, implementation, outcome measurement and quality analysis [9,15,16]. Decades of employment research on the communication of roles and responsibilities, has also shown that ambiguity and vagueness in role descriptions negatively impacts on service user expectations and outcomes, job satisfaction and staff turnover, intra-organisational collaboration, and personnel job performance [17,18,19].

In 2013 the Australian Government launched the National Disability Insurance Scheme (NDIS). The NDIS provides individualised funding for health and social supports to eligible people with a disability. To be eligible the person must have a permanent disability, that significantly affects their ability to take part in everyday activities and under 65 years old. The direct funding to the Scheme participant enables them to choose, coordinate and purchase the available services that meet their needs [20,21]. Each participant undergoes a planning process including setting their own goals, from which reasonable and necessary services are costed and submitted for approval. The NDIS recognised the need for CM in its policy and operationalisation, and embedded CM in its structures, although the role is called by other names [22,23]. Every participant in the NDIS has a case manager involved in the goal setting and planning process for funding. Once funding is allocated, further CM services may be provided to the participant although the specific CM actions vary depending on the needs (e.g. service availability, participant supports), and funding allocated for CM [24]. The various case manager roles in the NDIS provide a critical participant support to navigate the system, have choice and make decisions on, and coordinate support services, and service providers [25].

Our study used a top-down nested hierarchical content-analysis of components in CM role descriptions. We examined official documents and job positions of the case managers in the NDIS in New South Wales (NSW). The aim was to answer the following questions:

– Is the NDIS case manager job, role/s, and actions (interventions) clearly and consistently communicated in key documents (governance documents; operational guidelines; general (service) information; job descriptions; job advertisements)?
– Has the role/s and action description changed over time (from the pilot, roll- out and finalisation of implementation)?
– Is there vagueness, ambiguity, or gaps in the descriptions of the role/s and interventions of case managers?

In ***Table 1*** there is an outline of the implementation stages of the NDIS and CM job titles, employer organisations and geographic areas.

**Table 1:** Implementation stages of the NDIS and CM job titles.


	PHASE 1 PILOT STAGE 2013–2016	FULL IMPLEMENTATION STAGE 2017–2019

CM jobs	Local Area Coordinator (LAC) Support coordinator (for Disability Supported Accommodation only)	**2017** 1. Planner (office based) * 2. Local Area Coordinator (LAC) 3. Support coordinator **2019 additional jobs** 4. Specialist connection 5. Specialist support coordinator

Employer organisations	– NDIS (In NSW, the Ageing Disability and Home Care continued CM for people not yet a participant in the NDIS)	– St Vincent de Paul Society – Uniting (Uniting Care) – Social Futures *Planner – the NDIS
	Support coordinator funded through participant’s plan	The Support coordinator, Specialist connection and Specialist support coordinator are funded through participant’s plan

Areas	Four states (out of seven states/territories) with pilot sites In NSW (Hunter and Nepean/Blue Mountains)	All Australian states/territories


## Methods

We analysed documents from the Australian Government and one state, New South Wales (NSW). The reasons are NSW was one of the four (of seven) states/territories in Australia in the pilot; national implementation occurred over years; NSW implemented the NDIS state-wide early: NSW has the highest population thus more people employed in NDIS related CM jobs.

In this paper we refer to the: *job* as the paid case management position; *role* as the function and purpose of the person employed in the job; and *actions*, which are the interventions/activities performed by the person in the job.

We conducted a content analysis of the publicly available official and primary source documents detailing CM jobs, role, and actions in the NDIS national context and specifically in NSW from the period 2013–2019. We used a top-down nested hierarchy and selected informative documents including NDIS reference documents and real-world information documents of case manager’s job requirements, the role, and actions.

### Content analysis approach

We adapted Mayring’s Qualitative Content Analysis Procedural Model to analyse texts and draw realistic conclusions [26,27] (refer to first two columns in Table 2). The approach enables the identification of variations in trends, similarities, differences, and gaps in the communication within documents. It differs from other qualitative text analysis methods such as thematic or evaluative analysis or quantitative methods (counting frequency of words), and ensures the level of detail, rigour, and interpretation required for the analysis of vagueness and ambiguity. Refer to ***Table 2*** for the description of the two phases and steps for the content analysis. We used a stepped level of segmentation for analysis (Step 3 of both phases).

**Table 2:** The two phases of content analysis and steps.


STAGE 1: PILOT AND ROLL OUT OF NDIS (2013–2017)			

Adapted Mayring’s Qualitative Content Analysis Procedural Model*			

**CONTENT ANALYSIS PHASES**	**TASKS**	**DOCUMENTS**	**FURTHER INFORMATION**

***Step 1 Pre-determination of order and analytical units***	*Definition of the material and analysis of the situation of origi*n	Publicly available documents relating to the CM jobs in the NDIS including the LAC positions in NSW	Documents with an adequate definition of CM and/or description of the role or actions to be performed.

	*Formal characteristics of the material*	Document categories: governing legislation; operational guidelines; general (service) information; job descriptions; job advertisements	CM job: titles include local area coordinator; support coordinator; case manager; planner

	*Differentiation of sub-components and analytical units*	Document analysis: A stepped level of segmentation for analysis.	Key phrases of definitions and CM interventions/activities extracted and grouped into efficient sub-categories, thereby reducing the whole text into core contents

***Step 2 1st targeted document search***	*Search of organisational websites and screening for exclusions*	Document exclusions: Duplicates, documents not in English, documents published before 2012, Early childhood early intervention CM role (ECEI)	Document sources: NDIS; Ageing Disability and Home Care; St Vincent de Paul; Uniting, Social Futures

***Step 3 Content analysis***	*Intra-document search and screening for exclusions*	Document exclusion: inadequate content	Example: “the company hired a case manager”.

	*First level of content analysis – text extraction and semantic differentiation of components into sub-categories*.	components into the efficient sub-categories of semantically distinct phases in the text across documents.	Example: Assesses the participant or refers for assessment

	*Second level of content analysis Mapping the sub-categories to the CMTaxonomy main actions finalised by agreement (first two authors)*	The sub-categories identified in the text where mapped to the main action categories in the taxonomy	Example: Category of assesses the participant or refers for assessment – is mapped to the CMTaxonomy action of ‘holistic assessment’.

**STAGE 2: FULL IMPLEMENTATION OF NDIS (2018–2019)**			

***Step 2 2nd targeted document search***	*Search, exclusion, and content analysis*	Key organisational websites Exclusions: Documents with an adequate definition of CM and/or description of the role or actions to be performed.	Document sources: St Vincent de Paul, Uniting and Social Futures.

***Step 3 Content analysis***	*Intra-document search and screening for exclusions*	Compared to the 2017 documents, for new or missing phrases to be identified. Only new phrases to be analysed.	Inadequate descriptors in job advertisements and job description for support coordination positions

	*First level of content analysis – text extraction and semantic differentiation of components into sub-categories*.	Only new phrases were matched to efficient sub-categories	New phrase e.g., ‘gather information, implement, build and review plans’

	*Second level analysis – mapping to the CMTaxonomy main actions*		Not possible for support coordination (x3) jobs


* Mayring, P. (2014); Hsieh, H. F., & Shannon, S. E. (2005).

### Definitions

Ambiguity exists when a term can be interpreted in several ways. Vagueness occurs when the boundaries of the word’s meaning are not well defined, it is imprecise [28]. Both ambiguity and vagueness create uncertainty [29].

### Use of the taxonomy to assess vagueness and ambiguity

The definition and classification of “case-management” is a major problem in the ontology of integrated care in health and social sectors [9,10,15]. The problem relates to the high degree of ambiguity and vagueness of “case-management” as an integrated care strategy, as a type of support provided by a professional, as a modality of care, and as a personalised service within the cross sector care and social support system. Although ambiguity and vagueness have been the subject of considerable attention in general ontology and linguistics, these concepts have not been sufficiently explored and differentiated in the health care and social supports context, despite their significant detrimental effect on service delivery, costs of care, care quality, planning and management [29]. We used the CMTaxonomy, a validated framework used in policy, research, and practice. This framework provides a common language; identifies the main actions of CM, defines, and shows the relationship between key components of case management practice [2,16,30,31]. This study used the intervention tree main actions (parent category) to analyse whether ambiguity, vagueness and gaps existed. We identified a gap, when a critical CM role or action is absent in the position requirements. The level of detail in the documents available and analysed, did not allow analysis to sub-categories of actions (child categories) and related actions (grandchildren categories). Refer to Appendix 1 for the CMTaxonomy intervention tree. Refer to ***Table 3*** for the CMTaxonomy glossary definitions of the CM main actions.

**Table 3:** CMTaxonomy actions and definitions. Lukersmith, S (2017).


	MAIN ACTION	DEFINITION

1	Engagement	Establish, develop, and maintain a relationship with the client.

2	Holistic assessment	Evaluating the client’s health condition, functioning, environment, behaviour, situation or need for intervention; to develop a comprehensive understanding of them, their perspective, and what is important to them. Includes: their strengths, capacity, performance and needs across domains in relation to health, participation in key life areas (education, work, social, cultural and civic life), well-being and the contextual barriers and facilitators; assessment for the purpose of identifying appropriate intervention(s) and planning interventions. Excludes: monitoring

3	Planning	Supporting the person to develop their individualised plan including setting goals and priorities, actions, responsibilities to achieve the goals and identify the supports needed (services and resources).

4	Education	Providing structured information to person and stakeholders in a manner conducive to improve knowledge about matters relevant to the client’s health condition, medical, or rehabilitation treatment, functioning, situation, or strategies

5	Training and skills development	Teaching, enhancing or developing skills through context-specific practice to stakeholders. Includes: providing information or reinforcing training strategies developed by others for skill development e.g., memory or anger management strategies.

6	Emotional and motivational support	Providing the person (family and others as appropriate) with comfort, empathy or motivational support Includes: supportive communication (without using theory-based methods) to find strategies to solve or alleviate difficulties arising from their daily demands of life and situation; assisting, encouraging, and reinforcing the person (and family as appropriate) to build independence, make decisions, exercise choice and responsibilities, take actions, and support the client’s and family’s adjustment to changed circumstances

7	Advising	Recommending a course of action to be followed, to encourage a change of functioning, environment, attitude, or behaviour in relation to health, goals, or risks. Excludes: counselling and psychotherapy

8	Coordination	Navigating and facilitating the access, management, and cohesion of services and supports for the client.

9	Monitoring	Continuous acquisition of information to evaluate the client’s health condition, functioning, environment, behaviour, or situation over a defined period, to be able to determine their progress, anticipate or identify problems, additional goals or activities and modify plan and services as appropriate.


## Findings

We found ambiguity and vagueness in the CM definitions of the LAC from the employer partner organisations. There was a total of 28 unique definitions of CM roles in the NDIS documents and advertisements alone. We also found communication gaps on expected case manager actions across the various job descriptions for the same job but with different employers. There was poor role communication on the three support coordinator jobs with high variability in job title, job descriptions of roles and no adequate descriptions of actions required in these roles.

### Stage 1

#### Document search

**Figure 1:**
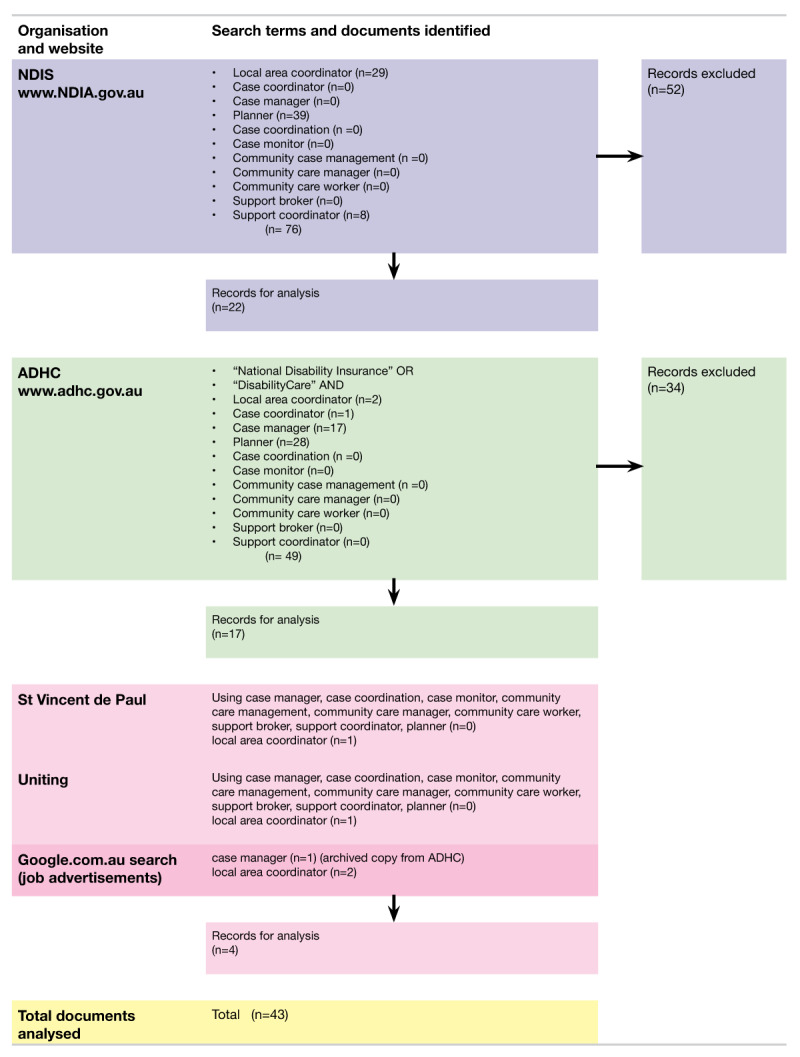
Search strategy and results.

***Figure 1*** provides the results from the internet search completed 6 – 20 March 2017.

Appendix 2 provides the references of documents analysed after exclusions from Stage 1 (n = 43) and Stage 2 (n = 10). The 2017 NDIS internal employee position of employed planner position description was not publicly available. There were no job descriptions for support connection, nor specialist support coordinator. ***Table 4*** provides the number of documents analysed per document category.

**Table 4:** Categories of analysed documents.


DOCUMENT CATEGORY	DOCUMENTS ANALYSED 2017	DOCUMENTS ANALYSED 2019

Governance documents	3	0

general (service) information	21	0

Operational/practice guides	14	2

job descriptions	2	7

job advertisements	3	3


#### Content Analysis

In Step 3 and the first level of analysis (refer to Table 2 phases of content analysis) there were 24 sub-categories of semantically distinct phases in the text across documents. ***Table 5*** lists the 24 sub-categories.

**Table 5:** Content extraction and analysis sub-categories.


SUB-COMPONENT CATEGORIES (N = 24)		

Provides support to implement plan	Develops goals for the next plan/reviews current plan	Assesses the participant or refers for assessment

Navigates, finds, connects, links with community supports	Investigates additional training/education options	Collates information to share with the team/completes compliance reporting

Provides technical support	Communicates any issues/barriers to stakeholders and implements solutions/strategies and problem solves	Educates external providers to enhance their ability to include individuals with disability

Explains how to identify options for further supports and priorities	Make decision/provides decision making support about needs and goals for completing plan	Coordinates the external services providers

Teaches how to enter service agreements	First point of person contact	Engages in activities to support ongoing development of the role

Maintains supports and has ongoing communication with participant	Discusses future long-term goals	Promotes inclusion and participation in the community

Monitors achievement of goals	Identifies risks and safeguards	Encourages changes in societal beliefs and disability

Explains the plan and provides information/answers questions	Assists with scheduling appointments and services	Advocates for human rights


The first two authors mapped the 24 sub-categories to the 9 main action categories in the CMTaxonomy until consensus reached. ***Table 6*** details the 24 sub-categories mapped to the CMTaxonomy main actions.

**Table 6:** Content analysis to CMTaxonomy categories – main actions.


	CMTAXONOMY MAIN ACTIONS								

SUB-CATEGORIES OF SEMANTICALLY DIFFERENT PHRASES (N = 24)	ENGAGEMENT	HOLISTIC ASSESSMENT	PLANNING	EDUCATION	TRAINING AND SKILLS DEVELOPMENT	EMOTIONAL AND MOTIVATIONAL SUPPORT	ADVISING	COORDINATION	MONITORING

Provides support to implement plan			✓	✓		✓	✓	✓	

Navigates, finds, connects, links with community, supports								✓	

Provides technical support					✓				

Explains how to identify options for further supports and priorities			✓	✓		✓	✓	✓	

Teaches how to enter service agreements					✓				

Maintains supports and has ongoing communication with participant			✓					✓	✓

Monitors achievement of goals									✓

Explains the plan and provides information/answers questions			✓	✓			✓	✓	

Develops goals for the next plan/reviews current plan			✓						

Investigates additional training/education options			✓				✓		

Communicates any issues/barriers to stakeholders and implements solutions, strategises and problem solves				✓		✓	✓	✓	

Make decisions/provides decision making support about needs and goals for completing plan			✓	✓		✓			

First point of client contact	✓								

Discusses future long-term goals			✓			✓			

Identifies risks and safeguards			✓				✓		

Assists with scheduling appointments and services								✓	

Assesses the participant or refers for assessment		✓							

Collates information to share with the team/completes compliance reporting								✓	

Educates external providers to enhance their ability to include individuals with disability				✓	✓				

Coordinates the external service providers								✓	

Engages in activities to support ongoing development of the role									

Promotes inclusion and participation in the community									

Encourages changes in societal beliefs of disability									

Advocates for human rights									


Below is a summary which outlines the inconsistency, vagueness, and gaps across documents. The summary starts with higher level of the hierarchy, the basic information (e.g., job title) and moves down the hierarchy to more detailed information on the job description of the role and then the case manager’s actions (interventions).

The job title used by the NDIS varied between documents with ‘local area coordinator’, (n = 15), ‘planner’ (n = 9) and ‘support coordinator’ (n = 2) creating ambiguity and confusion. We found 50 definitions (17 unique) in general NDIS documents and 16 definitions (11 unique) in NDIS job advertisements alone.

There were: four definitions (three unique) in two NDIS operational guidelines; 52 definitions (including 16 unique) in general information NDIS documents; 27 definitions (including 17 unique) in NDIS job descriptions; 16 definitions (including 11 unique) in job advertisements.

The most described sub-category in NDIS was ‘navigates, finds, connects, links with community and supports’ (n = 22 documents) located in operational guidelines; two operational documents; eight general information documents: one general document reporting on the ‘planner’ position; two general documents for ‘support coordinator’ role; job description and advertisements.

The CM sub-categories mostly commonly used (eight or more times) in NDIS documents: ‘navigates, finds, connects, links with community and supports’, ‘explains how to identify options for further supports and prioritise goals’, ‘maintains supports and has ongoing communication with participant’, ‘monitors achievement of goals’, ‘explains the plan and provides information/answers questions’, ‘develops goals for the next plan and reviews current plan’ ‘monitors achievement of goals’, ‘communicates any issues/barriers to stakeholders and implements solutions, strategies and problem solves’, ‘explains the plan and provides information/answers questions’ and ‘develops goals for the next plan/reviews the current plan’.

The most infrequent CM sub-categories or unique to a specific role included ‘educate external providers’ for the LAC role (NDIS guideline and St Vincent de Paul). In ‘LAC’ job position advertisements, the role of ‘promotes inclusion and participation in the community’ and ‘encourages changes in societal beliefs of disability’ was included.

Descriptions unique to NDIS support-coordinator in general information included ‘assists with scheduling appointments and services. Categories of activities used only in NDIS documents include: ‘provides technical support’, ‘teaches how to enter service agreements’, ‘educates external providers to enhance their ability to include individuals with disability’, ‘engages in activities to support ongoing development of the role’, ‘encourages changes in societal beliefs of disability’, ‘advocates for human rights’. These actions could not be mapped to the CMTaxonomy actions being actions in the domain of community development and business development, not individual case management.

In the pilot phase of NDIS, the terms ‘Case Manager’ and ‘Local Area Coordinator’ were used interchangeably [32,33]. The most used term was LAC, followed by planner and case manager, then support coordinator.

The LAC key responsibilities, and actions are the case manager’s role to support the participant. However, we found since 2013 LAC job descriptions also tasked them with community development of service providers with actions and responsibilities. Job descriptions included: “Contribute to building inclusive communities through partnership and collaboration with individuals and families/carers, local organisations and the broader community” (p.4 Candidate information pack 25 October 2013); ‘advocates for human rights’; ‘promote inclusion and participation in the community’; ‘encourage change in societal beliefs’; ‘educating external providers (to enhance their ability to include individuals with disability)’.

### Stage 2

Searches for job advertisements were on *google.com.au* search engine; organisational and employment websites (*au.indeed.com* and *Seek.com*) using key words of the CM job titles in June– August 2019. The content analysis involved analysing any change in the primary source documents of the CM jobs from 2017 to 2019 across the three partner organisations employing LACs in NSW. There was no change in job advertisements for LAC since 2017 except for St Vincent de Paul LAC job descriptions had one additional description e.g., ‘gather information, implement, build and review plans’ mapped to holistic assessment, planning and monitoring of the CMTaxonomy.

In February 2019, the NDIS expanded the positions for case managers from two to three levels of support coordination linked to costs. The NDIS briefly describes these roles:

– ‘Support connection: *assistance to implement their (*the participant) *plan*
– Support coordination: *assistance to implement their plan but including the design and build of their supports and linking to the broader systems across the complex service delivery environment…*
– Specialist support coordination: *assistance to implement their plan provided by expert or specialist approach necessitated by specific high complex needs…generally time limited (provided by an allied health professional)’*. (p. 52 [34]).

When the NDIS further revised these CM positions in July 2019, it was emphasised that:

‘*It is generally expected that participants will develop their capacity to implement and manage their supports and network more independently over time*’. (p. 33 [35])

Organisations external to the NDIS employ the people for these three support coordinator roles. There are NDIS approval processes for each category. Except for the brief descriptors above, there are no NDIS job descriptions. The employer develops their own job advertisements and descriptions. Internet searches for the support coordination and specialist support coordination roles produced a plethora of marketing, business promotion and advertising material with high variability in job titles and none or minimal job descriptions. There were ten job advertisements retrieved from different organisations for support coordination and specialist support coordination, but none located for support connection jobs.

Step 1 of the content analysis showed poor communication in job advertisements with significant variation and no consistency across the job titles which included: ‘support coordinator’; ‘coordinator of supports’; ‘case/package manager’; ‘case manager’; ‘coordinator/case manager’; administrative partner and NDIS support coordinator’; ‘case worker’; ‘coordination of supports’ (examples are direct quotes). There were less specialist support coordination advertisements compared to support coordination, although less variability in the title. The advertisements for these jobs often use the title of the potential employee’s qualification e.g., Occupational Therapist.

Two of the three support coordination jobs from February 2019 were new, thus there was no comparison over time of the job descriptions. Further most support coordinator advertisements lacked content on job description, descriptions of expected actions, applicant’s skill or personal requirements, rather there was a description of the organisational goals. Some examples are: ‘have a demonstrated understanding of the NDIS and experience managing funded plans’; ‘empowering NDIS participants to understand, implement and review their NDIS plans’; ‘have the skills to develop a systematic approach to assessments, care-plan reviews, monitoring; ensure that the services reach the expectations of the clients and introduce remedies to resolve any issues’; ‘report back to the NDIA on the outcome and goals, as required’. These job descriptions are generic, refer to personal attributes and display major communication gaps in what the support coordinator should do.

There is no description available of the case manager’s actions for post planning, coordination, implementation, and monitoring of support services in the participants plan. Inevitably this leads to confusion about expectations and responsibilities across the CM roles. Many participants (for example those with complex needs) have a LAC and support coordinator involved. We found many overlaps and ambiguity between different jobs and descriptions of actions. For example, navigating, finding, linking, and connecting is perceived as an action of three case manager jobs in the NDIS (the LAC, planner, and support coordinator), which creates confusion in responsibilities for both the workers and participant.

Planning and coordination actions are emphasised throughout, but there are major gaps. The case manager’s actions of engagement and holistic assessment are pre-planning actions fundamental to the planning with participants but are absent in the job descriptions up until 2019 [30]. The exception is communication of these pre-planning actions partially communicated in a 2019 job description of one partner organisations employing LACs. The support coordinator description includes “assists in preplanning activities’ but is not expected to undertake planning. Nor is it clear when assessment and pre-planning activities end and when actual planning starts.

## Discussion

Quality analysis and appraisal is critical to the future development of integrated care approaches and in particular a frequently used component, that of CM. To our knowledge, our study is the first analysis of the quality of the communication of the CM jobs, roles, and actions in a national disability system. We found there was ambiguity, vagueness and gaps resulted in poor communication of the role. We examined key documents on the roles of CM with the Australian NDIS from 2013–2019 with a focus on New South Wales. There are five CM roles funded in the NDIS Scheme.

The implementation of the NDIS has shown significant systemic problems impacting participant experiences in the planning, implementation and integration of their care [36]. Operational and quality challenges have arisen with case manager jobs and actions around person-centred planning and CM in the NDIS [1,2]. Various reports and enquiries on the NDIS report on participant’s confusion of CM role/s, expectations, responsibilities, concerns on the skill levels of case managers employed in the role, and outcomes of case manager actions [37]. The issues are reflected in the mounting quantitative evidence of 34% increase in NDIS planning and coordination support related legal reviews; participant appeals of decision made on plans appearing before the Administrative Appeals Tribunal; and the 400 complaints to the Commonwealth Ombudsman between 2016–2018 [20,22,38,39] and in NSW [21]. Ambiguity and gaps in communication on this critical NDIS case manager role and actions have contributed to participants confusion, expectations and poor experience of planning and outcomes. In part these failures and continuation of the issues led the Joint Standing Committee on the NDIS to establish an inquiry in July 2019 into NDIS implementation and performance issues [40] and another specific inquiry on case manager’s planning announced as recently as August 2019 [41,42]. The NDIS commenced a consultation process on support coordination with external stakeholders in August 2020 [43].

In the organisational psychology and occupational research literature role conflict and role ambiguity have been linked to employee burnout [44]. The more unclear the job description, the higher the role ambiguity, which leads to role conflict, occupational stress, and intention to quit [45,46,47]. Ambiguous and vague communication of work roles and actions also negatively affects job satisfaction, job performance and success, recruitment, role expectations and confusion around work tasks [48,49,50]. In contrast, consistent communication supports good governance, establishment of work standards for specific roles, quality appraisal and analysis, and workforce capacity building [51,52]. These are clear lessons for the CM roles for best practice integrated care including the NDIS sector and the impacts on the related workforce, of the current ambiguity and gaps in the poor role communication of CM.

The vagueness, ambiguity and gaps began in the pilot phase in 2013 and continue with further fragmentation with the recent creation of additional support coordination roles. Not all participants require or seek the same level of case manager support. CM jobs need opportunities to adapt their actions to meet local and participant needs appropriately for the context. However, it is clear from the mounting evidence, the ongoing ambiguity and gaps in the communication and lack of a shared vision on all CM NDIS roles, the expectations for actions to be performed by job incumbents has contributed to the variability in the operationalisation, quality, and outcomes for both the organisation and participant. Despite this, there has been little attention to the vagueness and ambiguity, and gaps in the communication of case manager jobs, roles, and actions, even though this is clearly a contributing factor to the current situation. The importance and need for an ontological approach with health and social care roles such as CM, which provides a common language, clear unambiguous communication on actions is well established [16,53]. This study highlights the need for an ontological approach to CM in the NDIS sector with greater attention towards quality and unambiguous communication on CM jobs and actions.

## Limitations

The study excluded one CM job in the NDIS, the ECEI. Parents typically perform some case manager actions in their role as parent of their children e.g., choice, assessment of need, support of the child and coordination of services. As the ECEI job role is different when compared to CM with adults, it was excluded.

We provide the reasons for including documents for only one state. There may be less ambiguity and vagueness in communication on the CM role and actions in different states, for example, if there is one partner organisation.

## Conclusion

Our study analysed the communication of CM in the context of the NDIS with a focus on NSW. The CM role is fragmented across various positions and there are gaps in the roles. The evolution into five different CM roles resulted in ambiguity and poor role communication. There is evidence through the numerous complaints and enquiries, of the impact of this on the quality and integration of care for participants of the Scheme. Future work may involve standardising the communication of the CM roles in the NDIS sector. An ontological approach to describing case management roles is needed. Such an approach uses a common language (such as a taxonomy), unambiguous and comprehensive communication at all levels and across industry related organisations to support best practice in policy, governance, workforce recruitment and capacity-building. The ontological approach would enhance the successful operationalisation of the NDIS and participant outcomes from planning and coordination. There are lessons on the importance of communication of a complex integrated care strategy such as CM communication for similar schemes in other countries and contexts.

## Additional Files

The additional files for this article can be found as follows:

10.5334/ijic.5590.s1 Appendix 1. Intervention tree (throughputs) of the community-based case management taxonomy.

10.5334/ijic.5590.s2 Appendix 2. Content analysis documents reference (2017).
