# Supplementary figures and images for: Vagueness and Ambiguity in Communication of Case Management: A Content Analysis in the Australian National Disability Insurance Scheme
Source: Int J Integr Care. 2021 Mar 19;21(1):17. doi: 10.5334/ijic.5590 (PMC7977023; doi:10.5334/ijic.5590)

# Intervention tree (throughputs) of the community-based case management taxonomy

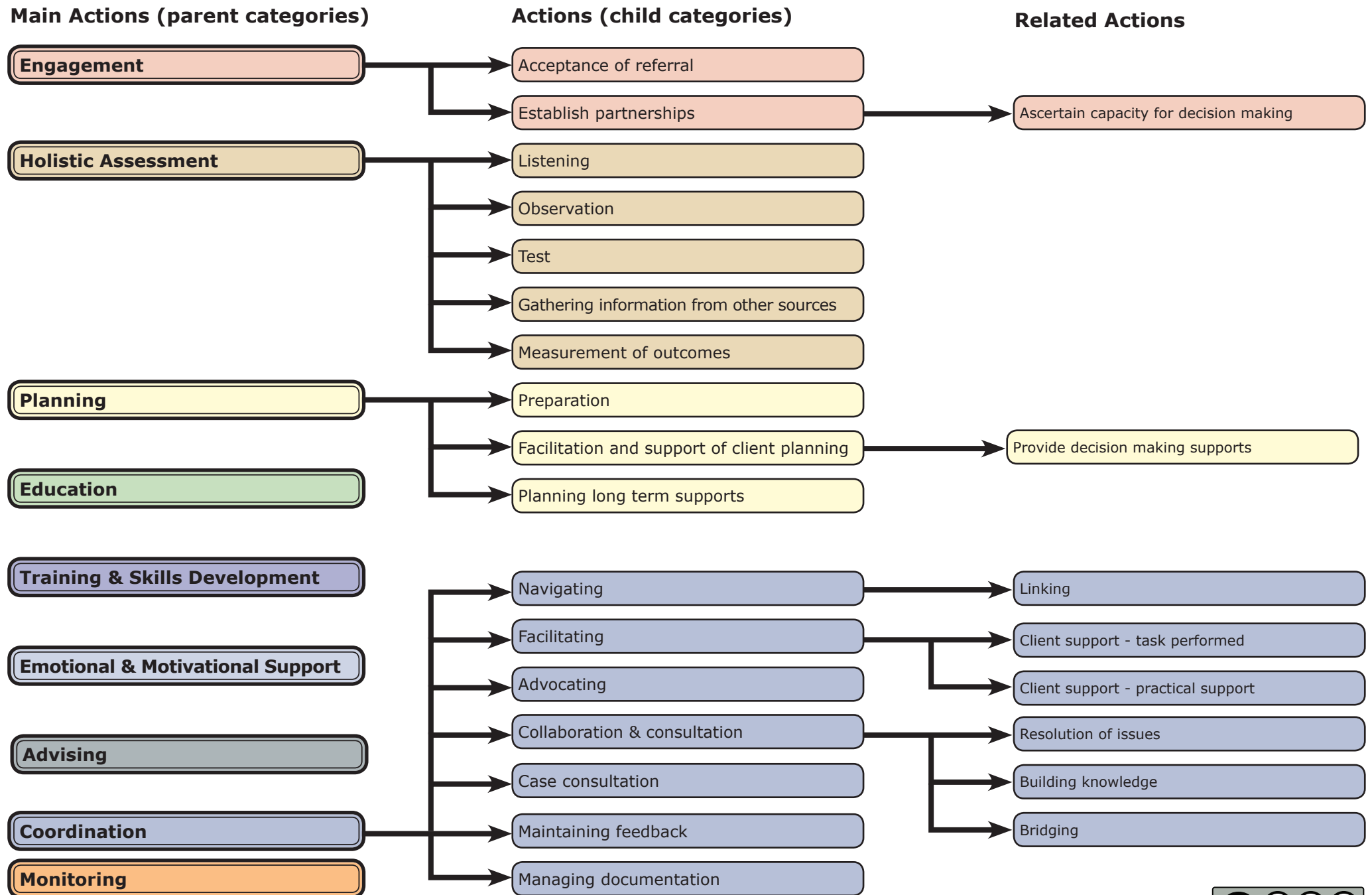

Supplement: Appendix 1. — Intervention tree (throughputs) of the community-based case management taxonomy. [file ijic-21-1-5590-s1.pdf]
