# Supplementary material for: Vagueness and Ambiguity in Communication of Case Management: A Content Analysis in the Australian National Disability Insurance Scheme
Source: Int J Integr Care. 2021 Mar 19;21(1):17. doi: 10.5334/ijic.5590 (PMC7977023; doi:10.5334/ijic.5590)
Supplement: Appendix 2. — Content analysis documents reference (2017). [file ijic-21-1-5590-s2.pdf]

Appendix 2

Content analysis documents reference (2017)

1. Bigby, C., & Douglas, J. (2015). Support for decision making - A practice framework. Retrieved from http://www.adhc.nsw.gov.au/__data/assets/file/0006/275685/Guidelines_NSW_CCSP_2015_v2.0.pdf
2. Gadow, F., & Riches, V. (2014). Practice guide to person centred clinical risk assessment. New South Wales Government, Family and Community Services,. Retrieved March 9, 2017, from https://www.adhc.nsw.gov.au/sp/delivering_disability_services/behaviour_support_services
3. National Disability Insurance Agency. (2017a). August 2015 enewsletter. Retrieved from https://www.ndis.gov.au/august-2015-enewsletter.html
4. National Disability Insurance Agency. (2017b). Consolidation and expansion. Retrieved from https://www.ndis.gov.au/consolidation-expansion.html
5. National Disability Insurance Agency. (2017c). Developing your child's participant statement. Retrieved from https://www.ndis.gov.au/participants/planning-process/developing-childs-plan.html
6. National Disability Insurance Agency. (2017d). Examples of services and support. Retrieved from https://www.ndis.gov.au/people-disability/examples-services-and-support.html
7. National Disability Insurance Agency. (2017e). Factsheet: supports the NDIS will fund in relation to early childhood. Retrieved from https://www.ndis.gov.au/document/factsheet-supports-ndis-will-fund-i.html
8. National Disability Insurance Agency. (2017f). Family support - what the NDIS will fund. Retrieved from https://www.ndis.gov.au/families-carers/family-supports.html
9. National Disability Insurance Agency. (2017g). FAQs - people with disability. Retrieved from https://www.ndis.gov.au/ILC-FAQ-People-with-Disability.html
10. National Disability Insurance Agency. (2017h). February enewsletter - we share some participant stories and seek your feedback. Retrieved from https://www.ndis.gov.au/february-enewsletter-we-share-some-parti.html
11. National Disability Insurance Agency. (2017i). IAC advice on reasonable and necessary support across the lifespan: An ordinary life for people with disability. Retrieved from https://www.ndis.gov.au/about-us/governance/IAC/iac-reasonable-necessary-lifespan.html
12. National Disability Insurance Agency. (n.d.-j). National Disability Insurance Scheme - NDIS home page. Retrieved March 9, 2017, from https://www.ndis.gov.au/
13. National Disability Insurance Agency. (2017j). National disability advocacy program. Retrieved from https://www.ndis.gov.au/participants/making-decisions-about-support/ndap.html
14. National Disability Insurance Agency. (2017k). NDIS features. Retrieved from https://www.ndis.gov.au/people-disability/videos-and-stories/cameos.html
15. National Disability Insurance Agency. (2017l). Operational guidelines - Overview of the NDIS. Retrieved from https://www.ndis.gov.au/operational-guideline/overview.html#5.1.1
16. National Disability Insurance Agency. (2017m). Operational guidelines - Planning. Retrieved from https://www.ndis.gov.au/operational-guideline/planning/timeframes-preparing-plan.html
17. National Disability Insurance Agency. (2017n). Part 3.1 - agency performance (cont.). Retrieved from https://www.ndis.gov.au/part3-agency-payments.html
18. National Disability Insurance Agency. (2017o). Planning. Retrieved from https://www.ndis.gov.au/operational-guideline/planning/participant-statement-goals.html
19. National Disability Insurance Agency. (2017p). Programme 1.1: reasonable and necessary care and support for participants. Retrieved from https://www.ndis.gov.au/programme-1-1.html
20. National Disability Insurance Agency. (2017q). Safeguards. Retrieved from https://www.ndis.gov.au/participants/safeguards.html
21. National Disability Insurance Agency. (2017r). Starting my plan. Retrieved from https://www.ndis.gov.au/participants/startingmyplan.html

22. National Disability Insurance Agency. (n.d.-s). Starting my plan. Retrieved March 9, 2017, from https://www.ndis.gov.au/participants/startingmyplan.html
23. National Disability Insurance Agency. (n.d.-t). Starting my plan with a local area coordinator (LAC). Retrieved March 9, 2017, from https://www.ndis.gov.au/medias/zip/documents/h9a/hbd/8799411109918/Starting-my-plan-with-a-Local-Area-Coordinator-LAC
24. New South Wales Government. (2012a). Living life my way. Putting people with a disability at the centre of decision making. Outcomes of statewide consultations. Retrieved from https://www.adhc.nsw.gov.au/__data/assets/file/0018/262530/Stage_3_consult_report_Aug2012.pdf
25. New South Wales Government, F. a. C. S. (2011). Living life my way. Putting people with a disability at the centre of decision making. Outcomes of statewide consultations. Retrieved from https://www.adhc.nsw.gov.au/__data/assets/file/0006/254832/PCA_Consultation_Report.rtf
26. St Vincent de Paul Society. (2017). Local area coordinator - position description. Retrieved March 9, 2017, from https://www.vinnies.org.au/icms_docs/237753_PD_-_Local_Area_Coordinator.pdf
27. Uniting Care. (2017). NDIS local area coordination. Retrieved March 9, 2017, from https://uniting.org/join-our-team/current-vacancies/join-our-team-LAC
28. St Vincent de Paul Society. (2017). Local area coordinator - job advertisement. Retrieved March 9, 2017, from http://lccqld.applynow.net.au/jobs/VIN287-ndis-local-area-coordinator-sydney
29. Uniting Care. (2017a). Local area coordinator - job advertisement. Retrieved July 6, 2017, from https://www.carecareers.com.au/job/community-jobs/70924-ndis-local-area-coordinator-uniting-western-sydney/
30. FACS. (2012a). New South Wales Government - Family and Community Services. (2012a). Addendum to the standards in action manual: women with disability. Retrieved March 9, 2017, from http://www.adhc.nsw.gov.au/__data/assets/file/0008/338948/addendum_standards_in_action_manual_women_with_disbility.pdf
31. FACS. (2015b). New South Wales Government – Family and Community Services. (2015b). Complex communication needs. Practice guide for speech pathologists who support people with disability. Retrieved March 9, 2017, from https://www.adhc.nsw.gov.au/sp/delivering_disability_services/core_standards/speech-pathology
32. FACS. (2015c). New South Wales Government - Family and Community Services. (2015c). Guidelines for NSW community care supports program. 2.0. Retrieved March 9, 2017, from http://www.adhc.nsw.gov.au/__data/assets/file/0006/275685/Guidelines_NSW_CCSP_2015_v2.0.pdf
33. FACS. (2016). New South Wales Government – Family and Community Services. (2015d). Individual funding handbook. Retrieved March 9, 2017, from https://www.adhc.nsw.gov.au/sp/delivering_disability_services/individual-funding
34. New South Wales Government - Family and Community Services. (2015c). Guidelines for NSW community care supports program. 2.0. Retrieved March 9, 2017, from http://www.adhc.nsw.gov.au/__data/assets/file/0006/275685/Guidelines_NSW_CCSP_2015_v2.0.pdf
35. FACS. (2013). New South Wales Government - Family and Community Services. (2013). Report on the trial of the national disability insurance scheme in the Hunter, New South Wales. 1. Retrieved March 9, 2017, from www.adhc.nsw.gov.au/__data/assets/file/0009/338814/Practice_Guide_to_Person_Centred_Clinical_Risk_Assessment.pdf

36. NSW Govt. (2012a). New South Wales Government - Family and Community Services. Living life my way. Putting people with a disability at the centre of decision making about their supports in NSW. Discussion Paper. Retrieved March 9 2017 from https://www.adhc.nsw.gov.au/__data/assets/file/0009/255663/ADHC_PCA_Stage3_DiscussionPaper.rtf
37. NSW Govt. (2012b). New South Wales Government - Family and Community Services. Living life my way. What you told us and what will happen next. A summary of the consultation process. Easy read version. Retrieved March 9 2017 from https://www.adhc.nsw.gov.au/__data/assets/file/0008/262907/Easy_English_consultation_outcomes_report.pdf
38. NSW Govt (2015d). New South Wales Government - Family and Community Services. (2015). Supports for children and young people 9-18 years and their families. Framework for service providers. Retrieved March 9, 2017, from http://www.adhc.nsw.gov.au/__data/assets/file/0006/332529/Supports_for_children_and_young_people_9_to_18_years_and_their_families_Framework_for_service_providers.PDF
39. NSW Govt. (2015a). New South Wales Government - Family and Community Services. (2015). Community participation. Choosing a service provider. Information for people with disability. Service providers in NSW. Retrieved March 9, 2017, from http://www.adhc.nsw.gov.au/__data/assets/file/0006/336543/20160901_CP_service_provider_booklet.pdf
40. NSW Govt. (2016). New South Wales Government - Family and Community Services. (2016). Individual funding handbook. Retrieved March 9, 2017, from http://www.adhc.nsw.gov.au/__data/assets/file/0014/320504/Individual_Funding_Handbook_Updated_version_2016_endorsed.pdf
41. NSW Govt. (2011). New South Wales Government - Family and Community Services. (2011). Living life my way. Putting people with a disability at the centre of decision making. Outcomes of statewide consultations. Retrieved March 9 2017 from https://www.adhc.nsw.gov.au/__data/assets/file/0006/254832/PCA_Consultation_Report.rtf
42. NSW Govt. (2017). New South Wales Government - Family and Community Services. (2017). What support may benefit you? Retrieved March 9, 2017, from https://www.adhc.nsw.gov.au/individuals/support/everyday_living_support/what_support_may_benefit_you
43. FACS. (2012b). New South Wales Government - Family and Community Services. (2012b). Case manager level 1 (identified) / Ageing Disability and Home Care (ADHC) Penrith. Retrieved March 9, 2017, from http://tradejobs.com.au/healthcare-and-medical-jobs/-case-manager-level-1-identified-ageing-disability-and-home-care-adhc-penrith/2262414

Documents referenced (2019)

1. Mid Coast Communities (June 2019) NDIS Local Area Coordinator Information package: Coffs Harbour NSW
2. St Vincent de Paul (June 2019) Local Area Coordinator Job advertisement and job description. Taree.
3. St Vincent de Paul (June 2019) Local Area Coordinator Position Description

National Disability Insurance Scheme (NDIS). (2016). *NDIS Price Guide VIC/NSW/QLD/TAS*. Geelong: NDIS.

4. Social Futures (July 2019) Local Area Coordinator Position advertisement file:///Volumes/SUZ%203_2019/Julia%20Good/Paper/health%20and%20social%20care/recent%20job%20ads%20and%20descriptions/Social%20Futures%20-%20Local%20Area%20Coordinator.webarchive
5. Social Futures (June 2018) Local Area Coordinator Position description Ballina
6. Uniting (2019) General description https://uniting.org/services/disability-services/uniting-and-ndis/local-area-coordinators
7. National Disability Insurance Scheme (2019) website broad description of the Local Area Coordinator role https://www.ndis.gov.au/understanding/what-ndis/whos-rolling-out-ndis/lac-partners-community
8. National Disability Insurance Scheme (2016) NDIS Price Guide VIC/NSW/QLD/TAS, Geelong, released 24 June 2016 https://www.ndis.gov.au/providers/price-guides-and-information/price-guide-archive
9. National Disability Insurance Scheme (2018/2019) NDIS Price Guide VIC/NSW/QLD/TAS, Geelong, released 1 February 2019 https://www.ndis.gov.au/providers/price-guides-and-information/price-guide-archive
10. National Disability Insurance Scheme (2019/20) NDIS Price Guide VIC/NSW/QLD/TAS, Geelong, released July 2019 https://www.ndis.gov.au/providers/price-guides-and-information
